# Ultrasound-Guided Percutaneous Tenotomy of the Long Head of Biceps Tendon in Patients with Symptomatic Complete Rotator Cuff Tear: In Vivo Non-contRolled Prospective Study

**DOI:** 10.3390/jcm9072114

**Published:** 2020-07-04

**Authors:** Luca Maria Sconfienza, Domenico Albano, Carmelo Messina, Salvatore Gitto, Vincenzo Guarrella, Carlo Perfetti, Ettore Taverna, Paolo Arrigoni, Pietro Simone Randelli

**Affiliations:** 1 IRCCS Istituto Ortopedico Galeazzi, 20161 Milano, Italy; io@lucasconfienza.it (L.M.S.); carmelomessina.md@gmail.com (C.M.); vguarrella@hotmail.com (V.G.); perfec@libero.it (C.P.); ettore.taverna@eoc.ch (E.T.); 2 Dipartimento di Scienze Biomediche per la Salute, Università degli Studi di Milano, 20133 Milano, Italy; sal.gitto@gmail.com (S.G.); pietro.randelli@unimi.it (P.S.R.); 3 Sezione di Scienze Radiologiche, Dipartimento di Biomedicina, Neuroscienze e Diagnostica Avanzata, Università degli Studi di Palermo, 90127 Palermo, Italy; 4 ASST Pini-CTO, 20122 Milano, Italy; arrigoni.p@gmail.com; 5 RECAP-RD, Università degli Studi di Milano, 20133 Milano, Italy

**Keywords:** shoulder, long head of biceps tendon, tenotomy, ultrasound, ultrasound-guided procedure, percutaneous, pain

## Abstract

Background: We prospectively tested technical feasibility and clinical outcome of percutaneous ultrasound-guided tenotomy of long head of biceps tendon (LHBT). Methods: We included 11 patients (6 women; age: 73 ± 8.6 years) with symptomatic full-thickness rotator cuff tear and intact LHBT, in whom surgical repair was not possible/refused. After ultrasound-guided injection of local anesthetic, the LHBT was cut with a scalpel under continuous ultrasound monitoring until it became no longer visible. Pain was recorded before and at least six months after procedure. An eight-item questionnaire was administered to patients at follow-up. Results: A median of 4 tendon cuts were needed to ensure complete tenotomy. Mean procedure duration was 65 ± 5.7 s. Mean length of skin incision was 5.8 ± 0.6 mm. Pre-tenotomy VAS score was 8.2 ± 0.7, post-tenotomy VAS was 2.8 ± 0.6 (*p* < 0.001). At follow-up, 5/11 patients were very satisfied, 5/11 satisfied and 1/11 neutral. One patient experienced cramping and very minimal pain in the biceps. Six patients had still moderate shoulder pain, 1/11 minimal pain, 2/11 very minimal pain, while 2/11 had no pain. No patients had weakness in elbow flexion nor limits of daily activities due to LHBT. One patient showed Popeye deformity. All patients would undergo ultrasound-guided tenotomy again. Conclusion: ultrasound-guided percutaneous LHBT tenotomy is technically feasible and effective.

## 1. Introduction

Complete rotator cuff (RC) tears are a common cause of disability and shoulder pain [1]. Treatment options depend on several factors, including the patient’s age, possible comorbidities, work activity, degree of disability and expected compliance with physiokinesis therapy after surgery. Conservative treatment has success rates ranging from 40% to 82% [2,3], although frequent complications such as reduction of subacromial space and progression of glenohumeral arthritis are reported in the literature [4]. Surgical treatment of complete RC tears may be the most appropriate choice [5,6,7]. However, although surgical RC repair generally guarantees satisfactory results, the rate of re-tearing can be up to 70%, particularly in cases of massive lesions and in patients over 65 years of age [8,9,10,11]. The long head of biceps tendon (LHBT) plays a key role in the natural history of RC injuries; degenerative LHBT lesions are often associated with RC tears [12,13], and it has also been widely demonstrated that degenerate, but continuous LHBT can cause pain in these patients [2]. Indeed, the LHBT is highly innervated by sensory sympathetic nerve fibers, making this structure a crucial source of shoulder pain [14]. However, the biomechanical contribution of LHBT to the shoulder remains to be clarified in some aspects, its spontaneous rupture in patients with RC pathology is commonly associated with pain relief [2]. For this reason, arthroscopically guided LHBT tenotomy or tenodesis are commonly practiced in cases where spontaneous rupture does not occur, besides often being part of arthroscopic repair of RC tendons [15,16]. These two surgical options for LHBT pathology have shown similar clinical outcomes [2,17], although tenotomy seems to provide earlier pain relief, despite at higher risk of cosmetic deformity due to prominent bump of the muscle belly (Popeye sign), while tenodesis tends to result in superior functional outcomes [18,19,20]. The tenotomy is indicated in painful shoulders with ruptured RC tendons in which conservative treatments (rest, rehabilitation, oral anti-inflammatory drugs, steroid injections) have failed, in patients with irreparable injuries and in those not willing to undergo surgery, since a non-negligible number of patients do not wish to undergo shoulder arthroscopic surgery due to their concern for negative outcomes [1,21].

Over the last years, ultrasound (US) has gained a crucial role both in the diagnosis and the treatment of musculoskeletal conditions [22], including articular, bursal and peritendinous injections, tendon procedures and nerve blocks around the shoulder, with some of these interventions being able to replace surgery [23,24,25,26,27]. US-guided procedures on tendons mostly involve the injection of drugs, lavage of calcifications and needling [28,29]. To date, there is still scarce evidence about the clinical value of percutaneous US-guided LHBT tenotomy. Some relatively recent studies on cadavers have shown its feasibility, allowing to quickly and completely sever the tendon with minimal skin incision [30,31,32], while Levy et al. raised some concerns regarding this procedure, highlighting the risk of iatrogenic tendon and cartilage injuries in cadavers [33]. Nevertheless, according to the last clinical indications published by the European Society of Musculoskeletal Radiology, this technique is already considered feasible, although not yet clinically tested [34]. The only reported case in clinical practice is by Greditzer et al. who illustrated the case of a non-operable patient, already subjected to RC repair, with severe LHBT tendinopathy, successfully treated by percutaneous US-guided tenotomy [35].

In this setting, a novel mini-invasive approach has been postulated to guarantee at least pain relief for those patients who have RC tear and still inserted LHBT. Thus, our purpose was to prospectively test the technical feasibility and clinical outcome of percutaneous US-guided LHBT tenotomy in vivo in patients with complete rupture of the RC.

## 2. Materials and Methods

### 2.1. Study Population

Our Institutional Review Board approved this prospective study and all patients provided their written informed consent to participate.

This study is concerned with the evaluation of patients subjected to US-guided percutaneous tenotomy of the LHBT at our Institution from March 2017 to September 2019. Sample size was calculated taking into account that previous literature on arthroscopic LHBT tenotomy yielded an improvement of shoulder pain of about 50%. Setting α = 0.05, this yielded a sample size of 11 patients. Sample size was calculated using G *power software (v. 3.1.9.2, Dusseldorf University, Germany) [36]. Patients were sent by different orthopedic surgeons after clinical evaluation and imaging examination (plain radiography and US and/or magnetic resonance). Inclusion criteria were i. informed consent signature; ii. being 65 years of age or older; iii. presence of symptomatic full-thickness tear of one or more RC tendons with intact LHBT confirmed at imaging; iv. patients not responding to conservative treatment (rest, physiotherapy, treatment with oral anti-inflammatory drugs, intraarticular/intrabursal injections, etc.); v. RC surgical repair not possible or refused. We excluded patients: i. with pseudoparalysis of the shoulder despite rehabilitative treatment; ii. with severe coagulopathies unable to stop anticoagulant therapy or allergy to local anesthetics; and iii. with pathologic conditions that contraindicate the tenotomy procedure, such as severe osteoarthritis or an acute inflammatory process in the periarticular soft tissues.

The visual analog scale (VAS) was used for pretreatment clinical assessment. It consists of a 10-cm graduated scale from 0 (no pain) to 10 (unbearable pain). Patients mark the point they believe best corresponds to their pain [37].

### 2.2. Tenotomy

Two radiologists with 15 years’ (L.M.S.) and 5 years’ (D.A.) experience in diagnostic and interventional musculoskeletal US performed the procedure. An ultrasound system (MyLab Seven, Esaote, Genoa, Italy) equipped with a high resolution linear array probe (SL1543, 13–3 MHz) was used. Prior to the procedure, a shoulder ultrasound was performed to confirm the RC tears and the presence of a continuous LHBT. The procedure was performed in a dedicated interventional room under strict sterile conditions with sterile cover for the US transducer and accurate skin disinfection using iodine-based solution [38]. The patient was placed in supine position with the arm of the affected shoulder along the trunk and the forearm in supination to obtain maximum LHBT traction [39]. The LHBT was identified with US in the rotator interval on the short axis as medial as possible. Local anesthesia was injected under US guidance (10 mL of 2% mepivacaine hydrochloride + adrenaline bitartrate 1:200.000) into the skin/subcutaneous tissue, around and within the LHBT. Then, a #11 scalpel was inserted on the anterior side of the transducer with an in-plane approach and advanced up to touch the anterior side of the tendon. The blade was positioned over the tendon to perform the cut under continuous US monitoring, with an anterior-to-posterior direction, until the LHBT was no longer visible [32]. The conclusion of the cutting was also confirmed by a “snap”, which was audible during the procedure and perceived by the operator under the scalpel. During the tendon cuts we ask the patient to flex the elbow against resistance to increase the LHBT traction. Then, the scalpel was removed, the skin cleaned with antiseptic solution and an adhesive skin closure (SteriStrip, 3 M, Milano, Italy) was applied. After the procedure, all patients were advised to take oral painkillers and to use local ice packs if needed in the days after the procedure. Patients were also instructed to remove skin closure autonomously after five days.

**Figure 1:**
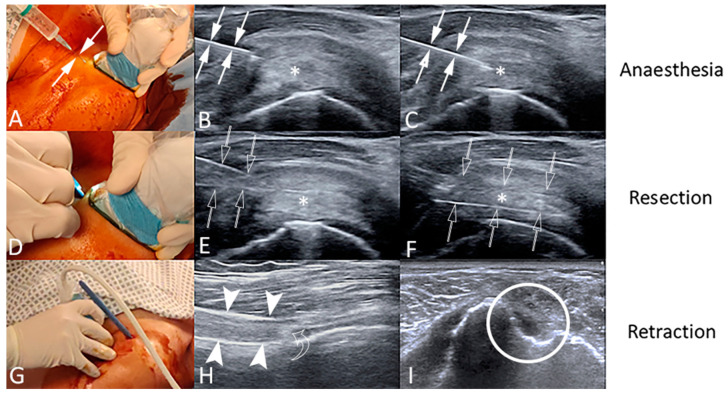
Ultrasound-guided percutaneous tenotomy of the long head of biceps tendon (LHBT) of a 69-year-old female patient. The procedure is performed under strict sterile conditions with sterile cover for the ultrasound transducer and accurate skin disinfection using iodine-based solution (**A**,**D**,**G**). The LHBT (asterisks) is identified with US on the short axis in the rotator interval as medial as possible. Local anesthesia is injected with a 23-gauge needle (arrows) under US guidance into the skin/subcutaneous tissue (**A**), around the LHBT (**B**) and within the LHBT (**C**). Then, a #11 scalpel (void arrows) is inserted on the anterior side of the transducer with an in-plane approach (**D**) and advanced up to touch the anterior side of the tendon (**E**). Then, the blade is positioned over the tendon to make the cut under continuous US monitoring, with an anterior-to-posterior direction (**F**), until the LHBT is no longer visible. Last, the US transducer is placed over the biceps groove (**G**) to demonstrate the technical success of the procedure, identifying the LHBT in long axis (arrowheads), with the retracted tendon stump (curved arrow) distal to the biceps groove (**H**) and the intertubercular groove resulting empty in short axis (**I**, circle).

We recorded the number of tendon cuts, the elapsed time from skin incision and scalpel retraction and the length of skin incision. Immediate complications were recorded. Technical success was defined as the ability of completing the tenotomy as initially planned according to our protocol [40,41], with the LHBT completely severed, recognized with US distal to the biceps groove, with the intertubercular groove resulting empty [32]. Figure 1 shows the different steps of the procedure performed in one patient of our series. Video 1 shows the procedure of LHBT tenotomy under ultrasound guidance (Video S1 as Supplementary Material).

### 2.3. Follow-Up

Technique efficacy was defined at a timepoint of at least six months as a pain reduction on the VAS scale of 5 points [40,41,42]. At least six months after the procedure, a physician from our institution, not included among authors and blinded to any clinical data, administered a list of eight questions to patients to understand patient-reported outcomes after tenotomy. The questionnaire was used in a previous study on arthroscopic tenotomy (Table 1) [42]. VAS evaluation was also repeated at this follow-up timepoint. Continuous variables were reported as mean ± standard deviation. Discrete variables were summarized as median and interquartile range. VAS scores before and after tenotomy were compared using the Wilcoxon signed-rank test, with statistical significance set at *p* < 0.001. The SPSS software (v. 26, IBM, Armonk, NY, USA) was used for calculation.

## 3. Results

Eleven patients (6 women, 5 males; mean age 73 ± 8.6 years; range 65–91) were enrolled according to our inclusion criteria. All patients presented with complete RC tear and continuous LHBT at the US examination performed on the day of the procedure. The procedure was carried on as planned in all cases, with the US proof of tendon retraction distal to the biceps groove in all patients. Technical success was 100%. A median of 4 (interquartile range = 4–4) tendon cuts were needed to ensure a complete tenotomy. The mean duration of the procedure was 65 ± 6 s (range 52–75 s). The mean length of the skin incision was 5.8 ± 0.6 mm (range 5–7 mm). No immediate complications related to the procedure occurred. At five days, one patient reported to our Department for increased shoulder pain related to fluid effusion in the subacromial bursa, which was then treated with US-guided steroid injection. At five and eight days, one diabetic patient reported to our Department for a delayed wound healing, which then resolved spontaneously. No patients were lost at follow-up. The mean follow-up was 10 ± 2.6 months (range 6–14 months). The mean VAS score was 8.2 ± 0.7 before the tenotomy and 2.8 ± 0.6 at the follow-up evaluation (*p* < 0.001). Technique efficacy was 100%. At follow-up, 5/11 patients were very satisfied (45%), 5/11 were satisfied (45%) and 1/11 was neutral (9%). Only one patient experienced cramping (about once per week) and pain (very minimal) in the biceps muscle. The remaining 10 patients (91%) did not complain of cramping or pain in the biceps. Six out of 11 patients (54%) had still moderate shoulder pain, 1/11 (9%) minimal pain, 2/11 (18%) very minimal pain, while 2/11 (18%) did no longer complain of shoulder pain after the procedure. None of the 11 patients had neither any weakness flexing their elbow nor limits of daily activities due to the biceps, while only one showed biceps muscle bulge (Popeye sign) although he did not mind it from a cosmetic standpoint. Further, 100% patients would undergo US-guided tenotomy again. Full patients’ clinical data and answers to the questions are reported in Table 2.

## 4. Discussion

Our main findings are that US-guided percutaneous tenotomy of the LHBT is a procedure with 100% technical success and technique efficacy, high degree of patient’s satisfaction and no immediate complications.

This prospective study is the first cases series in living patients. Looking at the technical side of the procedure, previous feasibility studies on cadavers tested different approaches to sever the LHBT. In the first series published by Levy et al. [33], the authors cut the intra-articular portion of the tendon with an anterolateral superior skin incision, 1 cm lateral and inferior to the coracoid, similarly to ours, but obtaining a successful tenotomy in only 25% of cases. However, the authors used an out-of-plane approach that can limit the efficacy of a procedure that needs to be very precise. Then, the procedure was done by a sonographer with unreported experience in image-guided interventional procedures. Further, the authors tried to cut the tendon intraarticularly, close to the anchor, a zone which is usually not well accessible using US. This point was also underlined in the cadaveric study by Sconfienza et al. who, indeed, preferred to perform the tenotomy in the most cranial part of the intertubercular groove with 100% success [32]. Aly et al. [30] performed the tenotomy on cadavers at the rotator interval using different arthroscopic scalpels, obtaining a complete tenotomy in 67% of cases. The last cadaveric study was published by Atlan and Werthel, who differently demonstrated the efficacy of an endoscopic backward cutter using a posterior arthroscopic portal [31]. In the only one case reported on living patient, the authors used a #10 scalpel for skin incision and an arthroscopic trigger finger release hook knife to cut the extra-articular portion of the LHBT between the distal edge of the subscapularis tendon and the proximal edge of the pectoralis major tendon [35]. Their patient experienced pain relief and was able to return to normal level of activity, with the proximal tendon stump still placed at the proximal portion of the intertubercular groove at follow-up US examination. In our study, we preferred to do an anterosuperior skin incision in order to cut the LHBT at the rotator interval as medial as possible and with an in-plane approach to continuously monitor the scalpel advancement. Our choice was dictated by several reasons. Indeed, despite the poor success rate of Levy et al., the rotator interval can be easily scanned by moving the patient arm [22,39] and the in-plane approach helped us to precisely cut the tendon reducing the risk of iatrogenic injuries. Cutting the LHBT as medial as possible allowed us to keep at minimum the length of the proximal tendon stump, which may potentially impinge inside the joint. In addition, we avoided the distal approach tested by Greditzer et al. [20] to decrease the risk of damage to the crossing fibers of the subscapularis tendon and to the recurrent branch of the circumflex artery of the humerus.

Our procedure had very high overall patient satisfaction rate, with 91% of patients being satisfied or very satisfied at follow-up examination. Only one patient was neutral and was also the only one presenting Popeye deformity, cramping once per week and very minimal pain in the biceps muscle. Furthermore, all patients experienced pain relief and would have their percutaneous tenotomy again, with neither weakness in elbow flexion nor limitation in daily activities due to the biceps. These results are in line with those reported by Meeks et al. who administered the same questionnaire to 104 patients subjected to arthroscopic biceps tenotomy [42]. Ninety-one percent of their patients were satisfied/very satisfied, 95% would have the tenotomy again, 13% had the Popeye deformity, and about 20% presented cramping once per week and very minimal pain in the biceps muscle. The low frequency of Popeye deformity could be related to our approach. Indeed, we cut the LHBT as medial as possible into the rotator interval thereby decreasing the risk of both Popeye sign and intra-articular impingement of the proximal tendon stump. On the other hand, we can postulate that a high percentage of arm fat could have left invisible the biceps deformity. However, we have not available data regarding body mass index or other measures of body fat percentage. We also found similar results in terms of pain relief to those reported in a recent study aimed to compare arthroscopic tenotomy and tenodesis [18]; indeed, the authors reported pre-tenotomy mean VAS of 7.5 (which was about 8 in our series) and post-tenotomy mean VAS of 4 (which was around 3 in our series). In this regard, it is important to highlight the main differences between arthroscopic tenotomy and our novel procedure. Arthroscopy needs to be performed in operating room with a team composed of orthopedist, nurse and anesthesiologist. It requires two skin incisions and regional anesthesia, it is a more-invasive and longer procedure. Conversely, US-guided tenotomy can be performed in dedicated interventional rooms or ultrasonography rooms and it involves a radiologist and a nurse. Further, it has shorter procedural time and requires only one mini-incision and local anesthesia.

No major complications were encountered, and the procedures were no painful with no significant bleeding. Indeed, although lidocaine is generally used for local anesthesia prior to US-guided musculoskeletal procedures [29,38], we preferred to use a solution of mepivacaine + adrenaline to reduce the risk of bleeding due to the relatively invasiveness of the procedure. Further, the percutaneous tenotomy led to a minimal skin incision and was very quick, requiring a mean time of about one minute from skin incision to scalpel retraction, in line with a previous feasibility study on cadavers where the mean elapsed time was 53 sec [32]. Notably, two minor complications were observed in our series. One patient complained of shoulder pain due to subacromial-subdeltoid bursitis, which was successfully treated with a single bursal injection of steroid. Moreover, we encountered a delayed wound healing in a diabetic patient, which then resolved spontaneously. It is well known that wounds can take longer to heal in patients with diabetes. Thus, we recommend reducing to a minimum skin incision paying particular attention to skin disinfection in case of uncontrolled diabetes in order to decrease the risk of delayed wound healing and infections.

Some limitations should be taken into account. First, our sample size was relatively small, although it was enough according to sample size calculation and it was sufficient to reach statistically significant results. Nevertheless, larger studies will enable to better understand the real frequency of complications related to this procedure. Then, this is a prospective non-controlled study, thus we did not compare the results of our procedure with those of arthroscopic surgery that is considered the standard of care. However, US-guided LHBT tenotomy was never tested before on living patients, thus a feasibility study was needed. We acknowledge that the shorter procedural time and higher cost-effectiveness of US-guided tenotomy do not require any comparison with arthroscopy. Nevertheless, future prospective randomized trial should be aimed at comparing our novel procedure to standard arthroscopy to understand the relevance of possible iatrogenic injuries to joint cartilage and RC tendons, as well as to assess the risk of intra-articular impingement related to the proximal LHBT stump and the clinical impact of possible missed diagnosis normally reached during arthroscopy. Last, we used a not validated questionnaire for follow-up clinical evaluation based on what previously published [42]. However, similarly to Meeks et al. we aimed at providing a biceps-specific questionnaire to assess clinical outcomes after tenotomy.

In conclusion, US-guided percutaneous tenotomy of the LHBT has 100% technical success and technique efficacy, high degree of patient’s satisfaction and no immediate complications. Future prospective randomized studies comparing the outcome of this procedure with the standard of care are warranted.

## Figures and Tables

**Table 1:** List of eight questions administered to all patients at least six months after the US-guided LHBT tenotomy.

**1. How would you rate your overall satisfaction with the surgery you had performed on your shoulder? On a scale of 1–5**				
1–Very unsatisfied	2–Unsatisfied	3–Neutral	4–Satisfied	5–Very satisfied
**2. Do you experience painful spasms or cramping in your biceps muscle? Yes or No If so how often per week?**				
1–More than five times per week	2–Five times per week	3–Once per week		
**3. Do you have any pain in the biceps muscle area? Yes or No. If yes, on a scale of 1–5**				
1–Very severe pain	2–Severe pain	3–Moderate pain	4–Minimal pain	5–Very minimal pain
**4. Do you have any shoulder pain? Yes or No. If yes, On a scale of 1–5**				
1–Very severe pain	2–Severe pain	3–Moderate pain	4–Minimal pain	5–Very minimal pain
**5. Do you have any weakness flexing your elbow, opening a can or using a screwdriver? Yes or No. If yes, which one?**				

**6. Does your biceps limit any of your daily activities? Yes or No. If yes, how limited are you?**				
1–Severely limited	2–Moderately limited	3–Minimally limited		
**7. Do you notice a biceps muscle bulge (“Popeye sign”)? Yes or No. If yes, do you mind it from a cosmetic standpoint? Yes or No**				

**8. If you could go back in time, would you have the surgery done again? Yes or No**				


**Table 2:** Full patients’ clinical data and answers to the questions.

Patient #	Sex	Age (years)	Questionnaire Item								Incision (mm)	Cuts (no.)	Duration (s)	VAS Pre-Procedure	Follow-Up (months)	VAS Post-Procedure
			1	2	3	4	5	6	7	8						
1	F	66	4	N	N	3	N	N	N	Y	6	4	63	8	6	3
2	F	71	5	N	N	N	N	N	N	Y	6	5	75	7	12	2
3	F	66	4	N	N	3	N	N	N	Y	7	4	67	8	8	3
4	M	67	5	N	N	5	N	N	N	Y	6	4	64	9	14	3
5	M	65	4	N	N	3	N	N	N	Y	5	3	69	9	10	3
6	M	91	4	N	N	3	N	N	N	Y	5	4	61	8	11	3
7	F	84	5	N	N	N	N	N	N	Y	6	4	52	8	7	2
8	M	70	5	N	N	4	N	N	N	Y	6	4	66	9	13	3
9	F	81	5	N	N	5	N	N	N	Y	6	4	68	7	9	2
10	M	76	4	N	N	3	N	N	N	Y	5	3	64	8	8	3
11	F	69	3	2	5	3	N	N	Y	Y	6	5	66	9	12	4
Mean/median		73 ± 8.6									5.8 ± 0.6	4	65 ± 5.7	8.2 ± 0.7	10	2.8 ± 0.6

Note: F = female; M = male=; N = no; Y = yes; Incision = length of skin incision; Cuts = number of tendon cuts to obtain a complete tenotomy; Duration = elapsed time from skin incision and scalpel retraction; no. = number.

## Supplementary Materials

The following are available online at https://www.mdpi.com/2077-0383/9/7/2114/s1, Video S1: Videoclip shows the procedure of LHBT tenotomy under ultrasound guidance.
